# From Young to Old: Mimicking Neuronal Aging in Directly Converted Neurons from Young Donors

**DOI:** 10.3390/cells13151260

**Published:** 2024-07-26

**Authors:** Nimmy Varghese, Amandine Grimm, M. Zameel Cader, Anne Eckert

**Affiliations:** 1 Research Cluster Molecular and Cognitive Neurosciences, University of Basel, 4002 Basel, Switzerland; nimmy.varghese@unibas.ch (N.V.); amandine.grimm@unibas.ch (A.G.); 2 Neurobiology Lab for Brain Aging and Mental Health, University Psychiatric Clinics Basel, 4002 Basel, Switzerland; 3 Department of Biomedicine, University of Basel, 4055 Basel, Switzerland; 4 Nuffield Department of Clinical Neuroscience, University of Oxford, Oxford OX3 9DS, UK; zameel.cader@ndcn.ox.ac.uk

**Keywords:** directly converted neurons from human fibroblasts, mitochondria, stress, cortisol, rotenone

## Abstract

A substantial challenge in human brain aging is to find a suitable model to mimic neuronal aging in vitro as accurately as possible. Using directly converted neurons (iNs) from human fibroblasts is considered a promising tool in human aging since it retains the aging-associated mitochondrial donor signature. Still, using iNs from aged donors can pose certain restrictions due to their lower reprogramming and conversion efficacy than those from younger individuals. To overcome these limitations, our study aimed to establish an in vitro neuronal aging model mirroring features of in vivo aging by acute exposure on young iNs to either human stress hormone cortisol or the mitochondrial stressor rotenone, considering stress as a trigger of in vivo aging. The impact of rotenone was evident in mitochondrial bioenergetic properties by showing aging-associated deficits in mitochondrial respiration, cellular ATP, and MMP and a rise in glycolysis, mitochondrial superoxide, and mitochondrial ROS; meanwhile, cortisol only partially induced an aging-associated mitochondrial dysfunction. To replicate the in vivo aging-associated mitochondrial dysfunctions, using rotenone, a mitochondrial complex I inhibitor, proved to be superior to the cortisol model. This work is the first to use stress on young iNs to recreate aging-related mitochondrial impairments.

## 1. Introduction

The investigation of aging has experienced a surge of interest in recent decades due to the increasing population of individuals aged 60 and over [1]. As individuals progress in age, their probability of encountering detrimental health outcomes and acquiring chronic conditions, such as neurodegenerative disorders, increases [2,3]. One of the prevailing beliefs is that stress experienced throughout a person’s lifespan is a primary cause of aging [4,5]. Stress, whether severe or chronic, can cause molecular damage that leads to age-related impairments [6]. As the primary regulator and mediator of stress is solely dependent on mitochondrial energy, the brain becomes one of the body’s most vulnerable organs during aging as adaptation to stress declines [7,8,9]. The main challenge in the field is to find easy-to-access, suitable, and reproducible in vitro models that mimic the human brain aging as accurately as possible. In the interest of using human neuronal material, the inaccessibility and ethical constraints make the usage of human brain material obtained during operations or postmortem challenging, thereby limiting neuronal aging research [10]. New approaches have emerged in recent years, such as generating human neuronal cells through direct conversion of neurons (iNs) from human fibroblasts (HFs) [11]. In our previous study [12], we showed that the iNs from HFs of aged donors displayed an aging-associated mitochondrial impairment compared to young individuals by demonstrating a similar phenotype as their cells of origin. By comparing iNs derived from young donors (age range: 24–36 years) and those from aged donors (age range 60–78 years), we observed that aged iNs showed decreased mitochondrial respiration, mitochondrial membrane potential (MMP), and cellular ATP levels. Moreover, aged iNs displayed a higher amount of mitochondrial free oxygen radicals compared to young iNs. To obtain a comprehensive bioenergetic profile, we further analyzed glycolysis profiles, revealing a metabolic switch toward anaerobic glycolysis in the aged iNs compared to the young. The aged iNs are a viable model system for studying human neuronal aging by maintaining an overall aging-associated donor phenotype. However, technological constraints are associated with using iNs from aged individuals, particularly when it comes to the limited cell number of neurons achieved [13,14]. The quantity of iNs is limited by the number of accessible HFs. Nevertheless, neurons taken from physiologically aged HFs may not reprogram as efficiently as those taken from younger individuals [13,14]. Furthermore, aged HFs are more prone to apoptosis during conversion due to sensitivity to environmental factors like temperature and pressure changes, hindering the generation of aged iNs. 

Stress approaches are commonly used to hasten pre-aging or pathological conditions in both in vivo and in vitro models to study alteration on the cellular and molecular levels [15], even extending to fibroblasts [16,17]. Experiencing stress is believed to trigger aging, leading to molecular and cellular alterations, including mitochondrial dysfunction, which further shapes the aging phenotype [18,19,20]. This paper aimed to establish a replacement model for aged iNs by subjecting young iNs to stress to mimic an aging-associated mitochondrial impairment. For this reason, acute stress approaches, either the human stress hormone cortisol as the “cortisol-model” or the complex I inhibitor of the OXPHOS system rotenone as the “rotenone-model”, were used to induce an aging-associated phenotype in young iNs. Since mitochondrial dysfunction is a crucial aspect of neuronal aging [21], our investigation focuses on mitochondrial bioenergetic properties. To the best of our knowledge, this study represents a pioneering effort to develop a “stress-model” as a neuronal aging model by employing iNs derived from HFs to mimic in vivo aging in a dish.

## 2. Materials and Methods

### 2.1. Chemicals and Reagents

Anti-PSA-NCAM-PE, human, mouse, and rat were purchased from Milteny Biotec. (Bergisch Gladbach, Germany) ß-tubulin III and NeuN were purchased from Abcam (Cambridge, UK). The plasmid pLVX-UbC-rtTA-Ngn2:2A:ASCL1 (UNA) was kindly provided by Salt Institute (San Diego, CA, USA) (now available on Addgene). The active Recombinant Human Noggin/NOG Protein was purchased from Abclonal Technology (Woburn, MA, USA), AZ960 by AdipoGen Life Science, and KC7F2 by APExBIO. Seahorse XF 100 mM Pyruvate Solution, Seahorse XF 1M Glucose Solution, and Seahorse XF DMEM Medium, pH 7.4, was purchased from Agilent Technologies (Basel, CH). Penicillin/Streptomycin and Puromycin 2 HCI (Powder) were obtained from Bioconcept (Allschwil, CH). Horse serum (HS) was purchased from Capricon (Ebsdorfergrund, DE). LDN-193189 and ZM 33637 were from Cayman (Ann Arbor, MI, USA). PBS was from Dominique Dutscher (Bernolsheim, FR). z-VAD-FMK was from Enzo Life Science (Farmingdale, NY, USA). All the following chemicals and reagents were purchased from Gibco (Waltham, MA, USA): B-27 Supplement (50X), DMEM/F-12, no glutamine, Geltrex LDEV-Free hESC-Qualified Reduced Growth Factor Basement Membrane Matrix, Gibco™HBSS, calcium, magnesium, no phenol red, GlutaMax, MEM Non-Essential Amino Acids Solution (100X) (NEAA), N-2 Supplement (100X), Neurobasal™-A Medium, and Tetracycline-free fetal bovine serum (FBS). Accutase was obtained from Innovative Cell Technology (San Diego, CA, USA). CHIR-99021 and Forskolin were obtained from LC-Laboratories (Woburn, MA, USA). SB-431542 and db-cAMP were acquired from MedChemExpress (New Jersey, NJ, USA). Recombinant Human/Murine/Rat BDNF and Human GDNF were obtained from PrepoTech. RQ1 RNase-Free DNase was from Promega (Madison, WI, USA). Laminin and NP-40 were from Roche diagnostics (Rotkreuz, CH). A83-1 was from Santa Cruz Biotechnology, Inc. (Dallas, TX, USA) Y-27632 (ROCK Inhibitor) was obtained from Selleck Chemicals (Houston, TX, USA). 2-deoxy-glucose, 3-(4,5-Dimethylthiazol-2-yl)-2,5-diphenyltetrazolium bromide (MTT), Antimycin A, Carbonyl cyanide-p-trifluoromethoxyphenylhydrazone (FCCP), DMSO, Doxycycline hyclate, Ethanol, Formaldehyde, Hydrocortisone, Myo-inositol, Oligomycin, Polyvinyl alcohol, Rotenone, Tetramethyl rhodamine, methyl ester, and perchlorate (TMRM), and 4′,6-Diamidin-2-Phenylindole (DAPI) were all obtained from Sigma Aldrich (St. Louis, MO, USA). BrainPhys was from STEMCELL Technology (Vancouver, B.C., CA). Dulbecco’s modified Eagle’s medium (DMEM), Dihydrorhodamine 123 (DHR), MitoSOX™ Red Mitochondrial Superoxide Indicator, Mitotracker Red CMXRos, TrypLE™ Select Enzyme were all purchased from Thermo Fisher Scientific (Waltham, MA, USA). Pyrintegrin was obtained from Tocris Bioscience (Bristol, UK). The commercial assay ATPlite 1step Luminescence Assay was acquired from System Perkin Elmer (Waltham, MA, USA) and MycoAlert^®^ PLUS Mycoplasma Detection Kit from Lonza (Basel, CH).

### 2.2. Cell Culture

#### 2.2.1. Primary Human Fibroblasts (HFs)

The primary human fibroblasts (HFs) and the corresponding directly converted neurons (iNs) were either obtained from Takara Bio (Kusatsu, Shiga, Japan) or were provided by Dr. Zameel Cader (University of Oxford) and predictive toxicology (StemBANCC) consortium. To access comprehensive donor information, refer to Table 1. The MycoAlert^®^ PLUS Mycoplasma Detection Kit was used to examine all cells regularly for mycoplasma.

The HFs were cultured in DMEM supplemented with 1% Penicillin-Streptomycin, 1% Glutamax, and 20% tetracycline-free FBS at 37 °C and 5% CO_2_ in a humidified atmosphere. For additional information, please see reference [12].

#### 2.2.2. Directly Converted Neurons (iNs)

**Figure 1:**
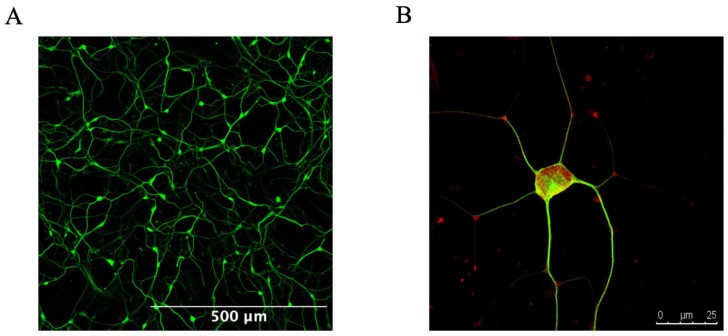
Representative images of young iNs 72 h post-FACS sorting. (**A**) Neuronal network of young iNs captured at 10× magnification, stained for ß-tubulin III (green). (**B**) High magnification (63×) view of a single neuron stained for ß-tubulin III (green) and NeuN (red). Both images were acquired using an inverted microscope (Leica Microsystems TCS SPE DMI4000). Abbreviation: iNs: directly converted neurons.

To generate the iNs, the methodology described by Zhou-Yang and colleagues was used, including minor modifications [22]. For the detailed protocol, please refer to [12]. In summary, the HFs were transduced with a puromycin-resistant lentiviral vector containing the ‘all-in-one’ vector pLVX-UbC-rtTA-Ngn2:2A:ASCL1 (UNA) in TMF medium. To induce neuronal conversion, UNA-transduced fibroblasts were pooled 3:1. One day after, the medium was replaced by neuronal conversion (NK) medium and exchanged every two days for up to 21 to 27 days. The iNs at this timepoint correspond to the immature neurons and are considered to have acquired a stable neuronal state and to be fully converted from fibroblasts to neurons. After the conversion, the cells were FACS sorted (Figure S1) by positive PSA-NCAM-PE (immature-neuronal marker) and negative DAPI (marker for dead cells) staining by using the FACS Sorter Aria III (FACS Core Facility, Biozentrum, University of Basel). By utilizing FACS, we achieved a relatively high purity of neurons. One day after FACS sorting, the medium was exchanged to neuronal maturation media and refreshed every day. After 72 h of plating, the neuronal connections could reform (Figure 1A,B), and measurements or stress approaches were conducted. At this timepoint, we also quantified the neuronal morphology (Figure S2). This allowed us to maintain a consistent cellular state for more accurate and reliable measurements of mitochondrial function. 

### 2.3. Experimental Treatment Design

**Figure 2:**
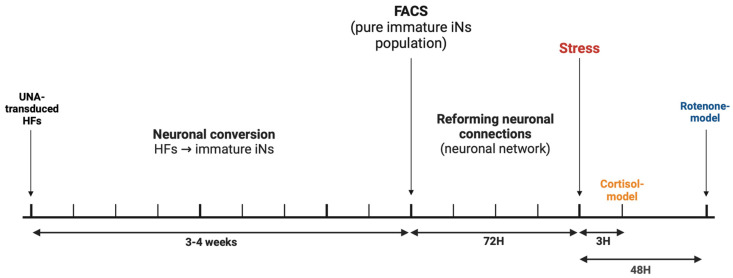
Experimental timeline. HFs carrying the ‘all-in-one’ vector UNA are converted to iNs over 3–4 weeks. The iNs were sorted using fluorescence-activated cell sorting (FACS) to obtain a pure population of immature neurons and then replated. After 72 h, the stress approaches were started. Abbreviations: HFs: human fibroblasts; iNs: directly converted neurons.

To investigate whether stress approaches are effective in mimicking cellular aging-in-a-dish, “stress” conditions were triggered either with cortisol (from 0.5 µM to 750 µM) or rotenone (from 2.5 µM to 25 µM) at different time points. Cortisol (Hydrocortisone) was prepared from a stock solution of 92 mM in ethanol, and rotenone was prepared from a stock solution of 25 mM in DMSO. The stress approaches were chosen based on our pre-screening experiments and are in line with previous studies using either cortisol [23,24] or rotenone [25,26]. The pre-screening experiments focused on determining the best stressor concentration and treatment duration by using the MTT and ATP readouts (see also the corresponding sections for detailed protocols). First, the MTT assays were performed to exclude the potential toxic treatment approaches at various concentrations after different exposure time courses (3 h, 24 h, 48 h, and 72 h). The ATP assay was further used to detect the best treatment approach to mimic an aging-associated energy metabolism in the cells (drop in ATP). The chosen stress approaches were a 20 µM cortisol administration for 3 h and a 10 µM rotenone stress approach for 48 h. Please refer to Figure 2 for the experimental timeline. Furthermore, the chosen stress approaches did not impact the cell viability of young iNs (Figure S3A,B).

### 2.4. MTT-Based Cell Viability Assay

Cell viability after stress treatments was detected using an MTT assay. The iNs were plated into clear 96-well cell culture plates [27]. The neurons were treated for 3 h with 5 mg/mL MTT. Afterward, the medium was wasted and 200 µL DMSO was added. After, the absorbance was measured at 570 nm with the Cytation 3 Cell Imaging Multi-Mode Reader. 

### 2.5. Total Cellular ATP Level

Cellular ATP concentration was quantified with the ATPlite 1step bioluminescence assay according to the manufacturer’s instructions [28]. The iNs were plated into white 96-well cell culture plates, and the bioluminescence signal was measured using the Cytation 3 Cell Imaging Multi-Mode Reader.

### 2.6. Mitochondrial Membrane Potential (MMP)

The fluorescent dye tetramethylrhodamine, methyl ester and perchlorate (TMRM) was used to measure the MMP [29]. iNs were plated into a black 96-well cell culture plate. Cells were incubated with the 0.4 μM TMRM dye for 30 min at 37 °C, 5% CO_2_ in a humidified atmosphere. Afterward, the iNs were washed twice with HBSS, and the fluorescence at 548 nm (excitation)/574 nm (emission) was detected using the Cytation 3 Cell Imaging Multi-Mode Reader (BioTek). The transmembrane distribution of the dye correlates to the MMP.

### 2.7. Reactive Oxygen Species (ROS) and Superoxide Anion Radical Levels 

The production of ROS and superoxide anions radicals were detected by using the fluorescent dyes dihydrorhodamine 123 (DHR, for mitochondrial ROS level) and MitoSOX (for mitochondrial superoxide anion radical level) [30]. The iNs were either incubated with 10 μM of DHR for 30 min or 5 μM of MitoSOX for 2 h at 5% CO_2_, 37 °C, and a humidified atmosphere. Following the incubation, the iNs were washed twice with HBSS. The conversion of the different dyes to their fluorescent derivate was detected using the Cytation 3 Cell Imaging Multi-Mode Reader at 485 nm (excitation)/535 nm (emission) for DHR and 531 nm (excitation)/595 nm (emission) for MitoSOX.

### 2.8. Mitochondrial Respiration and Glycolysis

Mitochondrial respiration and glycolysis were accessed using the Seahorse analyzers. The OCR and ECAR were measured using the Seahorse XF HS Mini Analyzer. Prior to measurements, the medium was exchanged to Seahorse XF DMEM medium, pH 7.4, and for OCR measurement, supplemented with 25 mM glucose, 4 mM glutamine, 1 mM pyruvate, and for ECAR measurements, with 4 mM glutamine and 1 mM pyruvate. The XF Mito Stress Test protocol (mitochondrial respiration) was performed according to the manufacturer’s instructions. The OCR rate was recorded under basal conditions followed by sequential injection of 2.5 μM oligomycin, 2 μM carbonyl cyanide-p-trifluoromethoxyphenylhydrazone (FCCP), and a combination of 2 μM antimycin A and 2 μM rotenone. The Seahorse XF Glycolysis Stress Test (glycolysis) was measured according to the manufacturer’s instructions by measuring the ECAR. The ECAR was detected under basal conditions, followed by 25 mM glucose, 2.5 μM oligomycin, and 25 mM 2-deoxy-glucose by sequential injection. The parameters were calculated manually. Additional information about the detected parameters and the exact calculations are described in [12].

### 2.9. Neuronal Morphology Quantification

In general, the iNs were fixed and stained with ß-tubulin III (1:100). The images were taken using an inverted microscope (Leica Microsytemes TCS SPE DMI4000) with a 10× objective. The images were analyzed using the NeurphologyJ interactive plugin (Eric Hwang Lab, Hsinchu, Taiwan, https://hwangeric5.wixsite.com/erichwanglab/neurphologyj, accessed on 26 June 2024) [31].

### 2.10. Normalization

Following the experiments, the iNs were stained with 4′,6-Diamidino-2-Phenylindole (DAPI) for 5 min and rinsed once with PBS. Next, the iNs were treated with 4% formaldehyde for 10 min and then rinsed twice with PBS. The iNs count was determined on the Gen5 Image 3.11 software. The iNs count obtained from a parallel running plate was used to normalize the ATP data.

### 2.11. Statistical Readout

Data management was performed using Excel and Graph Pad Prism 10 (version 10.2.3) software. 

## 3. Results

In our previous study [12], we were able to ascertain the aging phenotype in our aged iNs in comparison to our young iNs. Expanding on this work, we investigated whether stress-based methods on young iNs could potentially induce changes in the bioenergetic characteristics associated with aging, with a specific emphasis on the mitochondria. To visualize the effect of stress, we examined the established mitochondrial parameters on the cortisol and rotenone-exposed young iNs. To achieve our goal of replicating age-related mitochondrial impairments, we used the previously described aging profile of our HFs-derived iNs (Table S1) from aged donors as a reference for age-related alterations

### 3.1. Cortisol as a Potential Inducer of Aging in Young iNs Assessing Mitochondrial Properties

#### 3.1.1. Cortisol Model of Neuronal Aging Exhibited Bioenergetic Impairments in Young iNs

**Figure 3:**
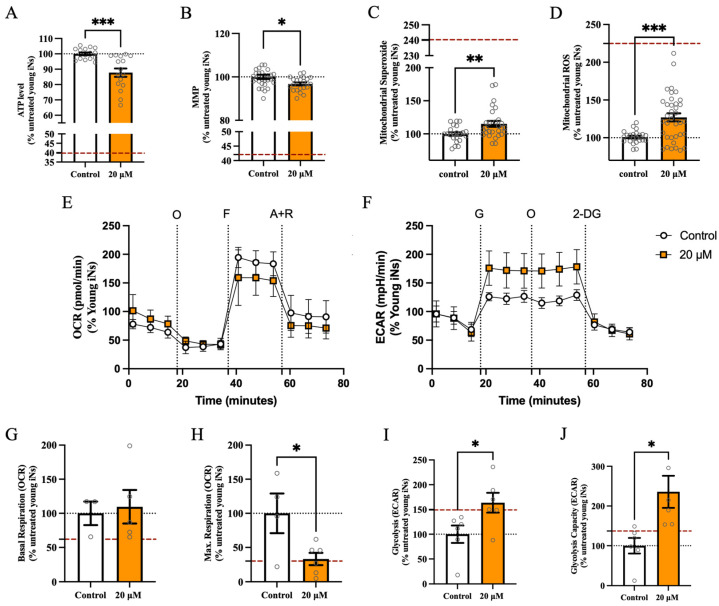
Cortisol exposure showed a trend toward bioenergetic impairment in young iNs but no effect on basal respiration. Assessment of (**A**) the total ATP content in the cell, (**B**) the MMP, (**C**) specific mitochondrial superoxide ion radical level, and (**D**) total mitochondrial ROS level indicated aging-related mitochondrial impairments. The real-time assessment of the Mitochondrial Stress Test (**E**) showed that cortisol did not affect the (**G**) basal respiration, but the (**H**) maximal respiration was decreased after cortisol exposure. The Glycolysis Stress Test (**F**) parameters showed an aging-associated rise in (**I**) glycolysis and (**J**) glycolytic capacity. Data show the mean ± SEM of 4 young donor iNs. The replicates of each donor are obtained from 4 to 11 independent experiments with *n* = 1–14 technical replicates per donor. All technical points are highlighted in the figure as circles. Values were normalized on the iNs count are shown as the percentage of the young iNs (=100%). The dashed black line represents 100% young iNs. The dashed red line represents the percentage of aged iNs to young iNs, as indicated in Table S1. Student’s unpaired *t*-test young iNs versus young cortisol-treated, * *p* < 0.05, ** *p* < 0.01, *** *p* < 0.001. Abbreviations: A + R: antimycin A + rotenone; ECAR: extracellular acidification rate; F: FCCP; G: glucose; iNs: directly induced neurons; Max: maximal; Mito: mitochondria; MMP: mitochondrial membrane potential; O: oligomycin; OCR: oxygen consumption rate; ROS: reactive oxygen species; 2-DG: 2-deoxy-glucose.

After the administration of cortisol, a notable decrease of 12% in the overall ATP level (Figure 3A) was observed compared to the untreated condition. Of note, this drop did not reach the degree of energy deficit associated with aging in iNs (dashed red line in Figure 1A, Table S1). Moreover, our findings revealed a statistically significant decrease in MMP levels (Figure 3B), by a marginal 3% compared to the untreated control condition. The levels of mitochondrial superoxide (Figure 3C) showed a statistically significant increase of 15% following the stress approach, in comparison to young iNs that were not treated. The level of ROS in the mitochondria (Figure 3D) exhibited a notable increase of 27% following exposure to cortisol, compared to young iNs that had not been exposed to cortisol. In the next step, we assessed the key parameters of mitochondrial respiration (representative OCR profile Figure 3E) and glycolysis (representative ECAR profile Figure 3F). While the basal mitochondrial respiration (Figure 3G) did not show a significant alteration after cortisol treatment compared to untreated young iNs, the maximal respiration rate (Figure 3H) significantly dropped after stressing with cortisol by 67%. Conversely, the glycolytic rate in the cortisol model showed an aging-associated increase in the critical parameters glycolysis (Figure 3I) by 64% and glycolytic capacity (Figure 3J) by 101%, by reaching the rate of aged iNs.

#### 3.1.2. The Impact of the Rotenone Model Revealed Aging-Associated Bioenergetic Impairments in Young iNs

**Figure 4:**
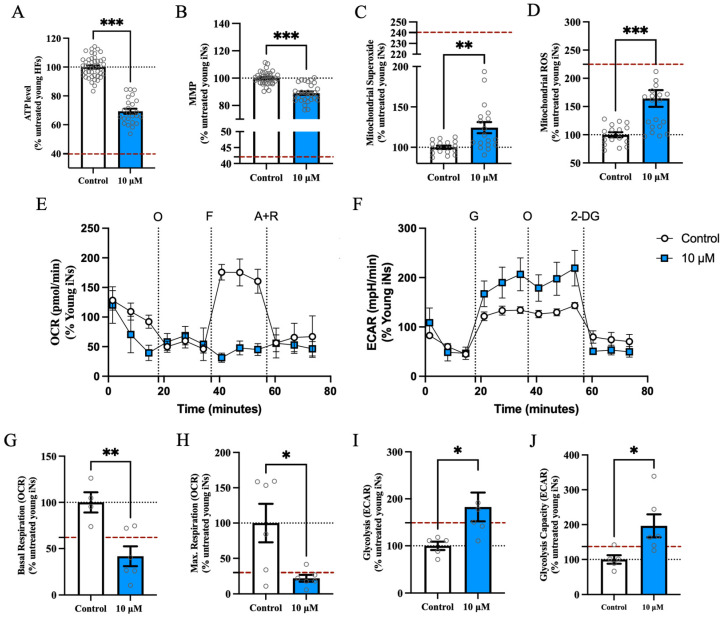
Rotenone-induced impairments on young iNs showed an overall aging-associated bioenergetic alteration. Assessment of (**A**) total ATP content, (**B**) MMP, (**C**) mitochondrial superoxide ion radical level, and (**D**) total mitochondrial ROS level revealed an aging-associated significant trend after rotenone exposure. Representative seahorse profile for Mito Stress Test (**E**) and Glycolysis Stress Test (**F**). The (**G**) basal respiration and (**H**) maximal respiration showed a decline in the rotenone model. The (**I**) glycolysis and (**J**) glycolytic capacity increased drastically after rotenone exposure. Data represent the mean ± SEM of 4 young donor iNs. The replicates of each donor are obtained from 4 to 11 independent experiments with *n* = 1–14 technical replicates per donor. All technical points are highlighted in the figure as circles. Values were normalized on the iNs count and are shown as the percentage of the young iNs (=100%). The dashed black line represents 100% young iNs. The dashed red line represents the percentage of aged iNs to young iNs, indicating the value from Table S1. Student’s unpaired *t*-test young iNs versus young rotenone-treated, * *p* < 0.05, ** *p* < 0.01, *** *p* < 0.001. All data represented in the figures are available. Please refer to the data availability section for more information. Abbreviations: A + R: antimycin A + rotenone; ECAR: extracellular acidification rate; F: FCCP; G: glucose; iNs: directly induced neurons; Max: maximal; Mito: mitochondria, MMP: mitochondrial membrane potential; O: oligomycin; OCR: oxygen consumption rate; ROS: reactive oxygen species; 2-DG: 2-deoxy-glucose.

After rotenone exposure, the total ATP levels (Figure 4A) significantly decreased by 31%. The MMP (Figure 4B) showed an 11% decline in the rotenone model. We observed a significant increase in mitochondrial superoxide (Figure 4C) and mitochondrial ROS (Figure 4D) after stress exposure between young iNs treated with rotenone and untreated condition, suggesting a heightened oxidative stress in the rotenone model. The impact of rotenone on respiration was evident in the mitochondrial respiration parameters (representative OCR profile Figure 4E), with a decrease in basal respiration (Figure 4G) after rotenone exposure by 58% and a significant decrease in maximal respiration (Figure 4H) of 78% to untreated young iNs dropping to the level of the aged iNs (dashed red line on Figure 4H). Furthermore, we observed via the Glycolysis Stress Test (representative ECAR profile Figure 4F) that glycolysis (Figure 4I) increased after rotenone exposure compared to untreated young iNs by 82%. The glycolytic capacity (Figure 4J) was significantly increased by 97%, exceeding the levels seen in untreated young iNs and surpassing those in aged iNs (Table S1). After stressing with rotenone, the bioenergetic state of young iNs showed an aging-related phenotype.

### 3.2. Comparative Analysis of Bioenergetics after Stress Exposure

**Figure 5:**
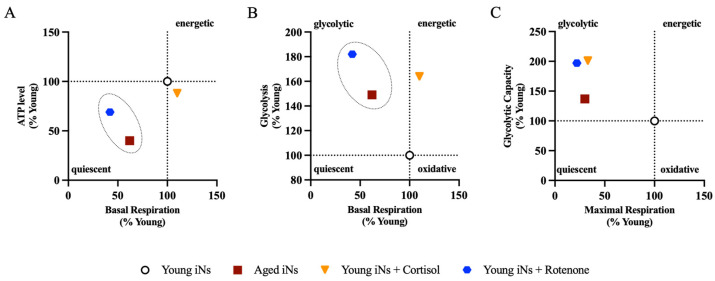
Comparative bioenergetic profiles representing aged iNs, cortisol model, rotenone model and untreated young. In (**A**), the basal respiration rate was plotted against cellular ATP to visualize energy depletion linked to the mitochondrial respiration. We compared basal respiration with glycolysis in the initial (**B**) bioenergetic profile. The graph (**C**) showed the energy map obtained by plotting the maximum capacity of extracellular acidification rate (ECAR) and oxygen consumption rate (OCR). The dashed areas/circles in (**A**,**B**) are drawn for visual purposes to cluster the rotenone model and aged iNs. These values/points represent the mean from the data of the previous figures or listed in Table S1. Abbreviations: ATP: adenosine triphosphate; iNs: directly induced neurons.

In a comparative analysis of our stress models on young individuals, we compared them to the aged individuals’ condition. The untreated young individuals are considered the baseline within all bioenergetic profiles, representing 100%. The aged iNs, on the other hand, correspond to the previously established values (Table S1). To examine mitochondria’s energy status under basal conditions, we plotted the levels of cellular ATP against the basal respiration state (Figure 5A). Rotenone was discovered to have a more pronounced effect on cellular ATP and basal respiration in iNs than the cortisol model. Cortisol was shown to have no impact on the basal respiration level. The bioenergetic phenotype was assessed by comparing the basal respiration with glycolysis (Figure 5B). The results suggested that the rotenone model had a glycolytic profile comparable to that of aged iNs. The cortisol model showed a change in their cellular state towards a more energetic metabolic profile, which differed from aged iNs. Finally, a bioenergetic profile was established by comparing the maximum capacity of mitochondrial respiration and glycolysis (Figure 5C). The OCR and ECAR parameters were explicitly chosen for direct comparison due to their statistically significant differences observed after the stress exposure. Both stress models indicated an aging-associated glycolytic energy profile at the maximal capacity level. Overall, the exposure to rotenone in young iNs was found to exhibit a more pronounced aging-related phenotype compared to the cortisol model. This significant finding is evidenced by a more substantial decrease in mitochondrial respiration along with an elevated glycolytic rate, underscoring the potential impact of mitochondrial stress on the bioenergetic profiles.

Our study revealed that the rotenone model exhibited a more significant aging-associated phenotype than the cortisol model (Figure 5A,B). When comparing the rotenone model with aged iNs, we observed a strong resemblance in mitochondrial ROS, respiration, and glycolysis (Figure 4D–H). However, the aging-related effect seemed less pronounced for ATP, MMP, and mitochondrial superoxide levels (Figure 4A–C).

## 4. Discussion

**Figure 6:**
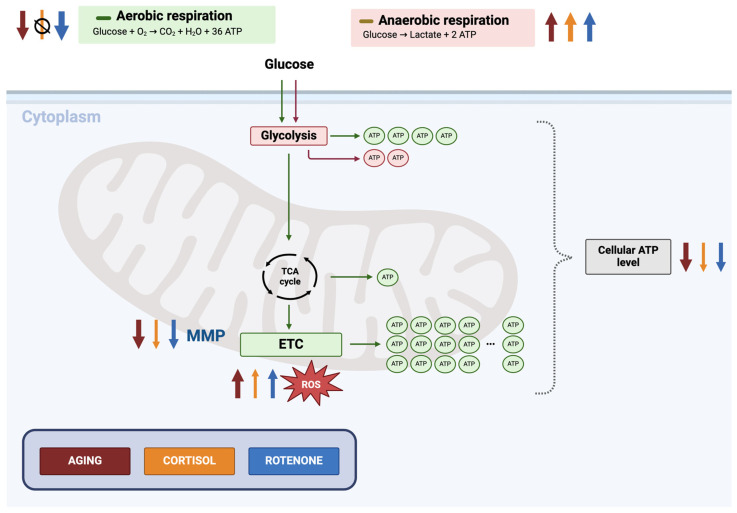
Schematic representation comparing the effects of cortisol or rotenone on young iNs and aged iNs compared to untreated young. According to our study, rotenone causes aging-related changes by decreasing mitochondrial respiration and increasing glycolysis and ROS levels. Cortisol, on the other hand, affects cellular ATP, MMP, and ROS levels but not mitochondrial respiration properties. The image simplifies the underlying processes for visualization purposes. The ↓ arrow shows a decrease and ↑ arrow represents an increase in the parameters. The red arrow indicates the aging phenotype, the orange arrow reflects the cortisol model of neuronal aging, and the blue arrow, the rotenone model of neuronal aging. The size or thickness of the arrows indicates the aging effect of the different conditions, whereas more prominent arrows indicate a more robust aging-associated phenotype. This image represents a simplified illustration of glycolysis and mitochondrial respiration. Abbreviations: ADP: adenosine diphosphate; ATP: adenosine triphosphate; CO_2_: carbon dioxide; H_2_O: water; MMP: mitochondrial membrane potential; ROS: reactive oxygen species; TCA: tricarboxylic acid. Created with BioRender.com.

Stress is considered to be the main trigger of aging. The main challenge in the aging research is to find easy-to-access, suitable, and reproducible models to investigate human brain aging and to find anti-aging drugs. The directly converted neurons of aged HFs are a promising tool in the aging field but are linked to limitations such as low reprogramming and conversion efficacy. In this paper, we aimed to establish a model system using young iNs stressed with either human glucocorticoid cortisol or mitochondrial complex I inhibitor rotenone to mimic an aging-associated phenotype and overcome the limitation of decreased availability of aged iNs. Our investigation centered on the bioenergetic impairments associated with mitochondrial dysfunction, a key hallmark of aging at the molecular level. We demonstrated that inducing age-related mitochondrial dysfunction through the inhibition of complex I with rotenone is superior to indirect approaches using cortisol (Figure 6). Rotenone triggered an overall aging-associated alteration on the bioenergetic state of young iNs, while the effect of cortisol was neglectable. Interestingly, cortisol was demonstrated to impair the mitochondria’s physiological “energy demand” state while showing no alteration under basal conditions. Overall, using the rotenone model appears to be a more promising model for studying human neuronal aging than the cortisol model.

### 4.1. The Human Glucocorticoids Induced a Partial Aging-Associated Phenotype

The implication of the human glucocorticoid cortisol in aging and pathological approaches is of great interest, mainly because the effects of psychological stress hormones on mitochondrial properties are poorly studied [32]. Elucidation of the effect of cortisol on mitochondria is an essential and uprising research area, especially in in vitro models. It is well-known that both cellular models—fibroblasts and neurons—express glucocorticoid receptors [33,34,35,36]. Unexpectedly and paradoxically, the cortisol-treated young iNs showed a reduction in the cellular ATP content, a decline in MMP levels, and a rise in oxidative stress by increased ROS levels compared to non-treated young. Despite significant variations, the trend associated with aging was quite unnotable. Our aging model of young iNs stressed with cortisol expressed an inconclusive alteration in mitochondrial respiration. Cortisol induced a pronounced decrease in maximal respiration in young iNs, whereas the baseline respiration rate appeared unaffected. Reciprocally, cortisol exposure increased the glycolytic rate in young iNs. 

Under normal conditions, cortisol is released during stressful situations to boost energy production to cope with the stress and to restore homeostasis [37]. Glucocorticoids (GCs), including cortisol, are thought to modulate mitochondrial function by interacting with GC-GR, either via the genomic or directly targeting the mitochondria, thereby affecting the expression of OXPHOS and mitochondrial proteins [38,39,40]. Prolonged or excessive doses of corticosterone could prevent mitochondrial energy production by inhibiting the electron transfer from NADH to the electron transport chain (ETC) through complex I in a non-specific manner [38,41,42]. This could explain the decrease in MMP and cellular ATP levels but not the lack of effect on bioenergetics. Studies have shown that glucocorticoid exposure affected mitochondrial function biophysically, depending on dose and duration [39], whereby short-term exposure to GCs could induce mitochondrial biogenesis and enhance enzymatic activity of selected subunits of the OXPHOS [43]. For instance, cortisol treatment of 1 h increased respiratory chain efficiency in isolated rat brain mitochondria, particularly in complex I-driven respiration [44]. However, prolonged and severe exposure to GCs caused increased ROS generation, abnormalities in mitochondrial structure, respiratory chain dysfunction, halted growth rate, and apoptosis [43]. For example, Abdanipour and colleagues found that chronic treatment impaired the growth and proliferation rate of neural stem/progenitor cells, which in turn led to apoptosis [45]. In general, long-term cortisol administration has a dynamic negative impact on major functional indicators of mitochondria, such as mitochondrial oxidation, Ca^2+^ levels, and MMP in neurons [37,38]. Even excessive cortisol treatment could negatively impact mitochondrial health. For instance, in the HT22 neuronal cell line from the hippocampus of mice, severe exposure to 400 μM cortisol for 3 h resulted in a significant decrease in cell viability. This decrease in viability was characterized by a decline in MMP and an increase in ROS levels [24]. Moreover, high and excessive levels of cortisol (>5 M) had dose- and time-dependent anti-proliferative effects on neuronal progenitor cells, further leading to apoptosis [45]. The decrease in maximal respiration observed in our cells may be due to cortisol exposure blocking their ability to respond to high energy demands rather than immediately affecting basal respiration. Indeed, if we had opted for prolonged and severe cortisol exposure, the overall mitochondrial respiratory capacity would show impairment, possibly intertwined with cell toxicity. Further investigation is required to evaluate the effect of cortisol on a molecular level. 

### 4.2. Rotenone Model of Neuronal Aging Is Superior to the Cortisol Model in Representing Mitochondrial Impairments Linked to Aging

Mitochondrial dysfunction is considered to be a hallmark of aging. Using a mitochondrial respiration inhibitor on young iNs could serve as a promising model to replicate aging-related bioenergetic deficiencies and intensify oxidative stress. Rotenone prevents the electron transfer from complex I to ubiquinone, inducing biomolecular abnormalities that mimic aging or neuropathological phenotypes [26,46,47,48,49,50]. Among mitochondrial enzymes, complex I activity is the most severely hampered ETC-unit since mtDNA encodes for seven complex I genes [51,52]. In our study, we found that rotenone exposure on young iNs caused mitochondrial respiration impairment, a metabolic shift towards glycolysis, and increased levels of ROS, reflecting a rise in oxidative stress. We observed that rotenone compelled young iNs to produce more ATP through glycolysis due to the shutdown of mitochondrial respiration, forcing the neurons to compensate for the energy deficit through higher glycolytic activity [53,54]. Observing a metabolic shift toward glycolysis induced by rotenone aligns with previous studies [55]. Using rotenone exposure accelerates the development of aging-related disorders by causing ATP deficiency and mitochondrial impairments. Cruz et al. demonstrated that exposure to rotenone-induced mitochondrial dysfunction led to an imbalance of superoxide and subsequent oxidative stress and DNA damage in HFs [16]. However, chronic use of rotenone-induced models could lead to significant neuronal degeneration and systemic toxicity, leading to cell death, which is a primary concern [56,57,58].

Our approach to mimic aging by utilizing stress as a trigger in young iNs showed that cortisol only partially reflected the alteration in neuronal bioenergetics associated with aging. Meanwhile, rotenone exposure mimicked an overall aging-associated bioenergetic profile in mitochondrial respiration, glycolysis, cellular ATP, MMP, and ROS levels. In our findings, the rotenone model was superior to the cortisol model for mimicking an in vivo aging-associated phenotype. The rotenone model can potentially be a valuable tool for conducting fundamental studies on the biology of aging at the mitochondrial level. It can assist in revealing novel concepts and mechanisms that contribute to broader aging research, such as the screening and development of drugs, hence facilitating the identification of new neuroprotective or anti-aging medications. Furthermore, the rotenone model might represent a promising cellular model for investigating pathological impairments, particularly in neuronal degeneration and behavioral defects associated with conditions such as Parkinson’s disease [56,57,58,59].

## 5. Limitations

In our experiments, we kept the iNs for 72 h in culture before starting the stress experiments. Typically, iNs in culture cannot be maintained over prolonged periods due to the inability of the culture environment to replicate the intricate conditions in the human brain fully [60,61]. These iNs cultures are often marked by visible signs of cell death after one week in culture after cell sorting. Extended cultivation of neuronal cells by up to two to three weeks is frequently achieved by co-culturing them with astrocytes, which support neuronal survival and function [62]. However, we opted to refrain from using astrocyte co-culture to maintain a more straightforward and cost-effective model system. To further investigate our stress models, particularly in a chronic setting, astrocyte co-culture systems should be employed to determine if a more pronounced aging phenotype can be observed. Moreover, prolonged cultivation combined with astrocyte co-culture generates mature neurons, allowing to observe the effects of stress on fully developed neuronal cells. This approach could provide deeper insights into the effect of chronic stress on neuronal aging and may present a better cortisol model. 

Another limitation is that our study primarily focused on assessing the functional bioenergetic properties of mitochondria. However, numerous additional investigational studies could be conducted to elucidate further effects of stress on cellular and molecular processes to mimic an aging-associated phenotype. For instance, future research could explore mitochondrial network morphology, providing insights into the structural changes. Moreover, investigating epigenetic and gene expression profile changes would be highly valuable. Such studies could reveal how stress exposure alters DNA methylation patterns and gene expression levels, providing a more comprehensive understanding of the molecular mechanisms underlying our stress models and how closely these models mimic aging. 

## 6. Conclusions

Taken together, these findings indicated that the tested stress approaches (cortisol or rotenone) showed potential for mimicking aging in vitro, especially on the mitochondrial properties. Aging is a chronic multifactorial process. Therefore, it might be argued that acute stress interventions alone are insufficient to elicit a comprehensive aging-related phenotype. The impact of the cortisol model only partially reflected an aging-associate decline in the mitochondrial bioenergetics, making short-term exposure to cortisol not effective enough. To mimic mitochondrial impairment linked to in vivo aging, direct inhibition of mitochondrial respiration by rotenone is superior to the cortisol model of neuronal aging. Our investigation, representing the pioneering effort to simulate an aging-associated phenotype in mitochondria by stressing young iNs, was met with considerable challenges. In conclusion, the rotenone model of neuronal aging holds significant potential as a viable strategy for exploring large-scale approaches as a replacement model for in vivo aged neurons. 

## Figures and Tables

**Table 1:** Donor information on the human fibroblasts (HFs). M: Man; Y: Year.

Identifier or Catalog Number	Age	Gender	Source
Cellartis^®^ Fibroblast P11031-C12	24 Y	M	Takara Bio
Cellartis^®^ Fibroblast P11019-C18	32 Y	M	Takara Bio
Cellartis^®^ Fibroblast P11028-C22	32 Y	M	Takara Bio
SF841	36 Y	M	The Cader Laboratory

## Data Availability

The data that support the findings of this study will be openly available after the peer-review process.

## Supplementary Materials

The following supporting information can be downloaded at: https://www.mdpi.com/article/10.3390/cells13151260/s1, Figure S1: Purification of iNs via FACS; Figure S2: Neuronal morphology quantification; Figure S3: Cell viability assay of the cortisol and rotenone treatment approaches. Table S1: Descriptive statistics of age-related changes in aged iNs compared to young iNs.
