# Supplementary material for: From Young to Old: Mimicking Neuronal Aging in Directly Converted Neurons from Young Donors
Source: Cells. 2024 Jul 26;13(15):1260. doi: 10.3390/cells13151260 (PMC11311457; doi:10.3390/cells13151260)
Supplement: Supplementary file 1 [file cells-13-01260-s001.zip › cells-3103315-supplementary.pdf]

Supplemental material

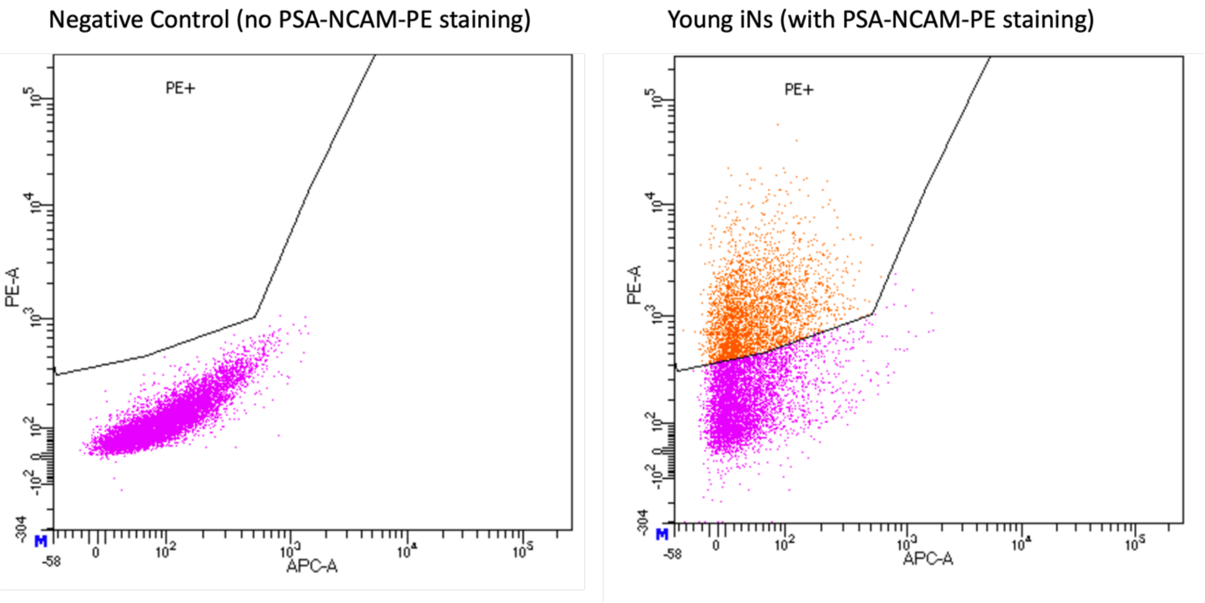

Supplementary Figure S1: Purification of iNs via FACS: FACS sorting was employed to separate neuronal cells from non-converted fibroblasts using the PSA-NCAM-PE (pro-immature neuronal marker). Typically, we run a negative control of iNs without PSA-NCAM-PE staining to define the gate for PE+ iNs. Based on this gating, we sorted our PE-positive iNs (in orange). This pure iNs population was then used for further investigation.

A

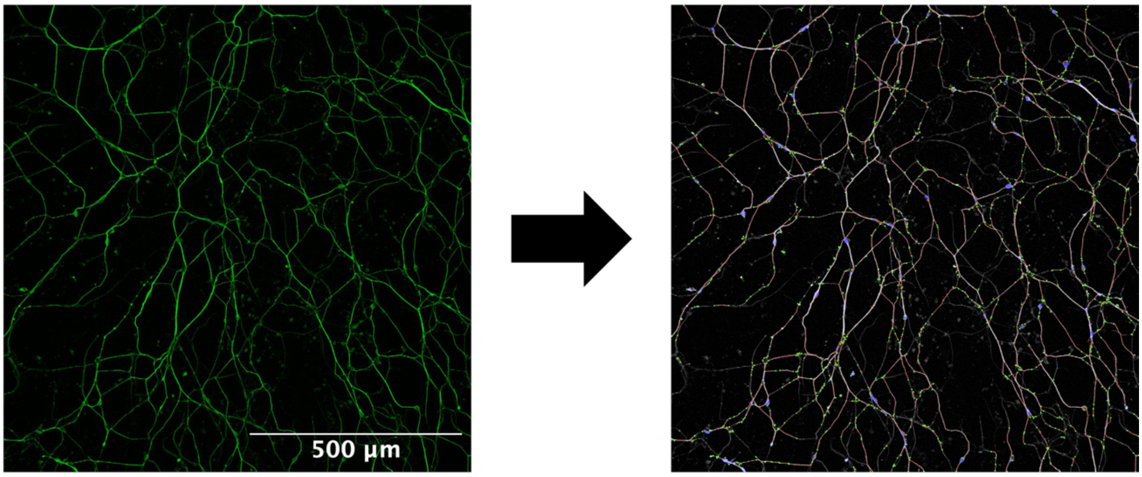

B

Slice	Amount
Soma count	83.6667
Neurite count	449.667
Neurite length (pixel)	19965
Number of branching points	206.333
Number of contact points	1061.33

Supplementary Figure S 2: Neuronal morphology quantification. (A) Representative image to represent the neuronal morphology quantification process using the protocol [1]. (B) represents a table presenting the output data derived from three independent experiments (N=3). This data table includes quantitative measurements of various morphological features, including the count of the soma, neurite, total neurite length, number of branching points, and number of contact points.

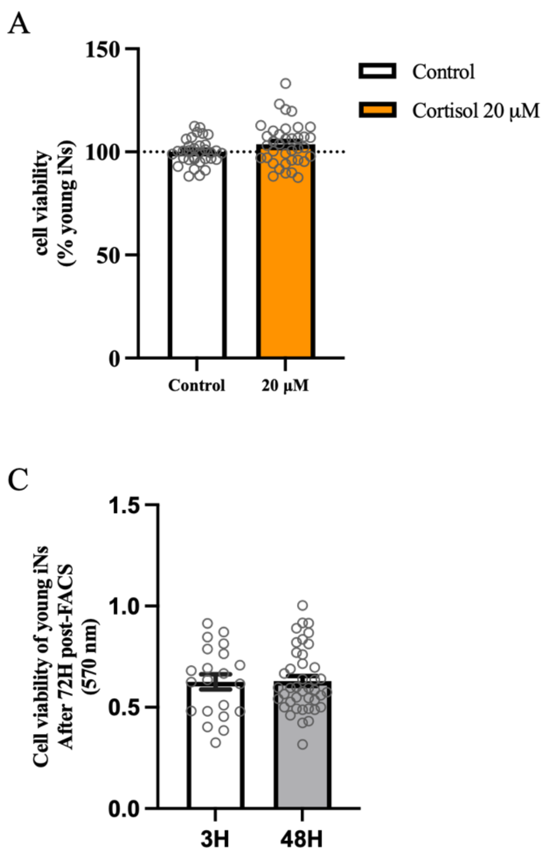

Supplementary Figure S3: Cell viability assay of the cortisol and rotenone treatment approaches.

Cortisol treatment approaches after (A) 3 hours, and rotenone treatment after (B) 48 hours on young iNs to determine the cell viability. The overall cell viability of untreated young iNs evaluated 72 hours post-FACS sorting, at different stress exposure time points is depicted in (C).

Supplementary Table S1: The table represents the descriptive statistics of study variables of the previous publication of our group [2] showing age-related changes in aged iNs compared to you young iNs. The ↓ - arrow (blue) shows a decrease and ↑ - arrow (yellow) represents an increase in the parameters in comparison to young. ATP: adenosine triphosphate, DHR: dihydrorhodamine 123; iNs: directly converted neurons; MMP: Mitochondrial Membrane Potential.

	**Young iNs**	**Aged iNs**
	**Percentage**	**Percentage compared to young (=100%)**
total ATP	100%	40% ↓
MMP	100%	42% ↓
Mitochondrial ROS	100%	225% ↑
Mitochondrial Superoxide	100%	240% ↑
Basal Respiration	100%	64% ↓
Maximal Respiration	100%	30% ↓
Glycolysis	100%	149% ↑
Glycolytic Capacity	100%	137% ↑

Reference

1. Ho, S.-Y., et al., *NeurphologyJ: an automatic neuronal morphology quantification method and its application in pharmacological discovery.* BMC bioinformatics, 2011. **12**: p. 1-18.
2. Varghese, N., et al., *Preservation of an Aging-Associated Mitochondrial Signature in Advanced Human Neuronal Models.* bioRxiv, 2024: p. 2024.03. 28.587193.
